# Social Health among German Nursing Home Residents with Dementia during the COVID-19 Pandemic, and the Role of Technology to Promote Social Participation

**DOI:** 10.3390/ijerph19041956

**Published:** 2022-02-10

**Authors:** Viktoria Hoel, Kathrin Seibert, Dominik Domhoff, Benedikt Preuß, Franziska Heinze, Heinz Rothgang, Karin Wolf-Ostermann

**Affiliations:** 1 Institute for Public Health and Nursing Research, University of Bremen, 28359 Bremen, Germany; kseibert@uni-bremen.de (K.S.); ddomhoff@uni-bremen.de (D.D.); wolf-ostermann@uni-bremen.de (K.W.-O.); 2 Leibniz Science Campus Digital Public Health, 28359 Bremen, Germany; rothgang@uni-bremen.de; 3 SOCIUM Research Center on Inequality and Social Policy, University of Bremen, 28359 Bremen, Germany; bpreuss@uni-bremen.de (B.P.); fheinze@uni-bremen.de (F.H.)

**Keywords:** COVID-19, social isolation, social participation, dementia, digital accessibility, social technology, nursing homes

## Abstract

The COVID-19 pandemic severely impacted the social health of nursing home residents with dementia due to social isolation. Consequently, the frequency of Behavioral and Psychological Symptoms in Dementia (BPSD) might increase. Technological solutions might help safeguard the social health of nursing home residents with dementia. This study investigates the impacts of the COVID-19 pandemic on clinical outcomes and the availability of social activities and technology to promote social participation in nursing home residents with dementia. The study analyzed cross-sectional data from a follow-up questionnaire nested in a larger national survey of care facilities in Germany. A mixed-methods approach integrated statistical analyses of closed-ended responses and thematic analysis of free-text responses. A total of 417 valid individual responses were received, showing an overall increase in observed BPSD—with anxiety and depression most frequently occurring. Many nursing homes canceled all social activities for residents with dementia, though a few had established procedures to facilitate social participation using technology. Requirements to promote social participation in this population using technology were identified at the micro-, meso-, and macro levels. Technology requirements permeated all three levels. During and beyond the COVID-19 pandemic, technology-driven solutions to promote social health among nursing home residents with dementia should be integrated into caregiving procedures.

## 1. Introduction

Since the World Health Organization declared COVID-19 a pandemic, country borders have been closed, and nationwide social distancing measurements have been implemented to curtail the spread of SARS-CoV-2 [1,2,3]. Among the vulnerable populations are people living with dementia (PLWD) due to aging, comorbidity and frailty [4,5,6,7,8]. This is especially true for PLWD in nursing homes, as their dependence on caregivers and healthcare providers reduces the opportunities for social distancing [1]. Nursing home staff are also exposed to a high risk of infection [9,10,11], not only due to the nature of their close-contact work but also the lack of availability of protective equipment and disinfectants during the first COVID-19 wave [12,13,14]. With this overhanging risk of infection, many nursing homes had to restrict visitation for family members and discontinue or reduce social activity offers for the residents [8,15,16,17].

Cognitive stimulation and social activities for PLWD are already limited in these settings, where research shows that nursing home residents spend as much as 65–85% of their time unoccupied, “doing nothing” [18,19,20,21]. Many nursing home residents with dementia are involved in few social activities in these facilities [18], having little interaction with others and spending time either alone watching television [22] or staying in bed for long periods [23]. This gives cause for concern, as participation in social activities–beyond routine primary and nursing care is an important indicator of the quality of life (QoL) in nursing homes [24]. Participation in social activities represents an essential building block of social health; a concept that emerged in the context of a debate on the definition of positive health [25,26]. Social health has been conceptualized as the influence of social and environmental resources in finding a balance between capacities and limitations, which relates to a person’s ability to adapt comfortably to different social situations [25]. The relevance of social health to dementia research has been promoted through the INTERDEM Social Health taskforce’s work in their spearheaded efforts in formulating directions for research and practice to promote social health in dementia caregiving [26,27]. Closely related to this, there is an increasing recognition of the importance of social participation for PLWD to meet their psychosocial needs and improve QoL [18,19,23,28,29].

However, psychosocial interventions might have been severely impeded by preventive measures against COVID-19 [2,8], increasing the social isolation of nursing home residents. A national pilot was recently conducted in a sample of Dutch nursing homes, where the existing visitation ban was lifted following national COVID-19 guidelines. Here, Verbeek et al. [17] found that the regained personal contact between nursing home residents and their relatives positively impacted residents’ well-being [17]. Conversely, this suggests that the increased isolation adversely affects the social health and well-being of nursing home residents [12], as evidence suggests that PLWD might exhibit problematic behavior as a response to unmet psychosocial needs [28,30,31,32]. This includes agitation, aggression, and depression, which are behavioral symptoms encompassed by the umbrella term “behavioral and psychological symptoms of dementia” (BPSD). For the purpose of this study, we utilize the BPSD definition proposed by Finkel and colleagues: “Signs and symptoms of disturbed perception, thought content, mood, or behavior that frequently occur in patients with dementia” [33] (p. 498). Long durations of unoccupied times and low levels of social participation have been found to contribute to worsening such behavior [8,23,30]. Although studies have shown that psychosocial interventions can be effective in decreasing BPSD [20,29,34,35,36], a large proportion of PLWD in nursing homes is treated with pharmacological therapy for behavioral problems [19,37,38]. However, psychotropic drugs demonstrate low effects on BPSD, with potentially substantial side effects [8,20]. Therefore, non-pharmacological interventions are recommended as first-line treatment for BPSD [34,37,38,39].

With limited social activities available and restricted visitation access for friends and families, technology is a promising non-pharmacological strategy to facilitate social participation for nursing home residents with dementia. To ensure social health for PLWD during the COVID-19 pandemic, according to recommendations from dementia association guidelines, technology has been emphasized as a viable way to improve mood and apathy and foster routines to treat anxiety among residents [8]. Furthermore, remote or simulated social interaction can allow residents to stay connected with friends and family, potentially reducing the feeling of isolation [2] and even decreasing agitation [40,41,42]. However, substantial barriers to such non-pharmacological strategies in nursing homes include the inability to provide infrastructure quickly, technology barriers, and sufficient staffing to support implementation [8,23]. In light of these challenges, this paper aims to assess the efforts put in place to safeguard the social health of German nursing home residents with dementia, as reported at the managerial level. The usage of technology to facilitate social participation among residents with dementia, and the prerequisites for doing so, were also explored. The following research questions guided the study:

1. Has there been an observable change in the clinical conditions of nursing home residents with dementia during the COVID-19 pandemic?
2. How did the COVID-19 pandemic impact the availability of social activities for nursing home residents with dementia?
3. How has technology played a role in ensuring social participation for nursing home residents?
4. What barriers and facilitators exist for people in need of care to use digital technologies for social participation?

## 2. Methods

### 2.1. Study Design

The present analysis is based on cross-sectional data from a follow-up questionnaire as part of a larger national online survey conducted in Germany during the second wave of the pandemic. The study reported here is part of a jointly designed study that includes outpatient and day center facilities, focusing on structural characteristics, the occurrence of SARS-CoV-2, and effects of the pandemic in terms of staffing, equipment and changed work processes and communication structures. These results are reported elsewhere [12,43]. The link to the follow-up survey was circulated via email among facility leaders and directors of nursing to an opportunity sample of 8187 German nursing homes throughout the country from 12 January to 7 February 2021. In addition, the survey was advertised through contacts of the study team to advocacy groups and provider associations. In advance, potential participants were provided with an information letter explaining the study and eligibility criteria. In the cover letter, employees from the management level (directors of nursing, managing directors, quality management officers and nursing staff acting as ward managers) were invited to participate in the survey. The questionnaire items were generated from internal project literature reviews and preliminary work by the study team, and the response time was approximately 20 min. The online survey was conducted using EFS Survey, Fall 2019 version (Questback GmbH, Köln, Germany, 2019).

The questionnaire items subjected to analysis for the aforementioned research questions contained closed questions (single or multiple choice) and open-ended questions (free-text format). In addition to the structural characteristics of the facilities and lab-confirmed cases of SARS-CoV-2, the survey included multiple sets of questions on the observed effects of the pandemic on nursing home residents with dementia, such as BPSD and increased use of pharmacological therapy. The question item inquiring about the occurrence of BPSD among residents with dementia provided a list of commonly occurring behaviors, including, but not limited to, depression, anxiety, apathy, hallucination, and wandering [8,33,44,45,46]. Efforts in maintaining social participation for nursing home residents with dementia were also inquired about, such as access to social activities, special visitation access and the establishment of procedures to utilize technology for social purposes. The amount of training provided for staff to implement and use technology in the facilities was also surveyed. Finally, participants also had the opportunity to make recommendations (in free-text) of requirements necessary to enable care recipients to use technology to promote social participation. A translated excerpt of the survey containing the question items subjected to analysis for the purpose of this study is available in the Supplementary Materials.

### 2.2. Data Processing and Evaluation

In a first step, participants were excluded who did not provide information on their care service/facility type (N = 360 respondents). The survey data of the remaining responses from nursing homes were included in the analysis. In the case of missing data, the facility was excluded from the evaluation for this item only. The evaluation was carried out descriptively using relative frequencies (of valid responses; N), mean values and chi-square independence tests (nominal significance level α = 0.05). An inductive thematic analysis approach was undertaken according to the thematic analysis guidelines described by Braun and Clarke [47] to analyze the free-text responses. As the online survey was conducted in German, results were initially compiled in German and then translated to English for publication. Primary translation was performed by the authors responsible for data collection and analysis and then validated by a third-party native speaker who had access to the German language results. Statistical analysis was performed using STATA version 12 software (StataCorp LP, College Station, TX, USA), while thematic analysis was performed using NVivo version 12 (QSR International Pty Ltd., Melbourne, Australia, 2020).

## 3. Results

### 3.1. Descriptive Statistics

A total number of 417 valid individual responses (around five percent of the invited facilities) were received, where most nursing home representatives worked in the nursing homes as facility managers (69.0%; N = 409) or directors of nursing (24.7%). The majority of facilities were non-profit (53.6%; N = 401) or private nursing homes (37.2%), while less than one in ten were public (9.2%). Of the respondents, 17.2% indicated that their facility had a special dementia care contract. The average number of healthcare professionals per facility was approximately 48, with an average client capacity of around 86 residents. Lab-confirmed COVID-19 cases among residents were reported by 212 (52.7%) of the surveyed nursing homes, with the average number of COVID-19 related deaths at around seven cases per facility since the outbreak (at the time of the survey), and 69.9% of the respondents reported lab-confirmed COVID-19 cases among their staff since the pandemic hit. In the following, results are reported in line with the research questions.

### 3.2. Clinical Conditions

There was an overall low increase in pharmacological therapy for nursing home residents with dementia, which was observed in less than six percent (5.6%; N = 344) of the facilities. Of the respondents, 87.5% had seen no increase, while the remaining valid responses were non-applicable. Summary statistics are listed in Table 1. However, when inquiring about observed BPSD among residents with dementia, a large proportion of the respondents saw an increase in at least one symptom, where depression (38.9%) and anxiety (38.6%) were most frequently reported. An increase in appetite loss (24.1%), aggression (16.9%) and wandering (16.9%) were also frequently observed among the survey respondents.

### 3.3. Social Activities for People Living with Dementia during COVID-19

More than a third (42.4%; N = 366) of the respondents reported that social activities for nursing home residents with dementia were canceled during the COVID-19 pandemic. In comparison, more than half of the survey facilities (56.0%) maintained their social activity schedule throughout the pandemic. A proportion of 14.8% (N = 284) of survey respondents reported that their nursing home granted special access for visitors of residents with dementia, while 85.2% did not. As Table 2 shows, no association was found between cancellation of social activities and nursing home structural characteristics, such as type of provider or whether the nursing home had a dementia care contract. Unsurprisingly, the cancellation rate of social activities for residents with dementia was correlated with whether the nursing homes had lab-confirmed COVID-19 cases among residents or staff. Staff shortages of more than 5.0% also showed a significant association with social activities for residents with dementia being canceled.

### 3.4. Promoting Social Participation for People Living with Dementia Using Technology

Less than seven percent (6.5%; N = 369) of German nursing homes had established procedures for using technology to promote social participation for PLWD. Nevertheless, when asked, “During the pandemic, did you create additional opportunities for care recipients with dementia to use digital technologies for social contact with friends, family or others?”, 72.8% (N = 349) reported on having done so, and 15.8% responded “No.” The remaining 8.3% planned to incorporate such procedures.

**Figure 1:**
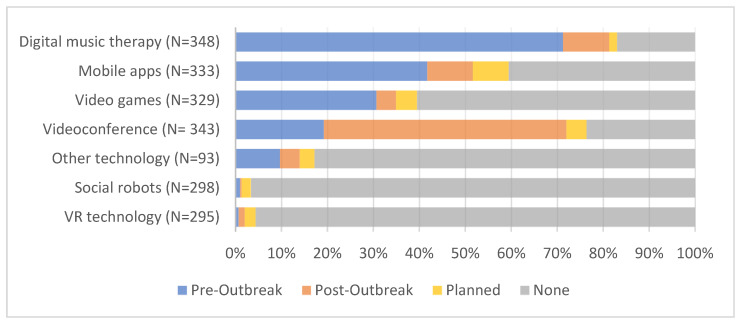
Utilization of digital devices to facilitate social participation for residents with dementia. Outlined are the relative frequencies of responses received per category. The pre-outbreak category includes the usage of post-outbreak (“Technology in use, even before the outbreak”).

As depicted in Figure 1, when inquiring about which digital device was being used to facilitate social participation for residents with dementia, there was an apparent preference for digital music therapy. The cumulative percentage (combined usage before and during COVID-19) was 81.3% (N = 348); however, 71.3% of the respondents already used digital music therapy *before* the outbreak. The same was true for mobile applications (41.7%; N = 333) and video games (30.7%; N = 329), which saw a modest increase during the pandemic (9.9% and 4.3%, respectively). The largest increase was seen, as expected, in the usage of videoconference tools. While 19.2% (N = 343) of respondents already used videoconferences to facilitate social participation for residents with dementia before the COVID-19 pandemic, 52.8% indicated that they first started after the outbreak. More novel technologies, such as social robots and VR technologies, had a low uptake both before and during the COVID-19 pandemic, accumulating to less than four percent of the total number of respondents.

The vast majority of respondents (50.7%; N = 353) had received *no* training in using technology to promote social participation among their care recipients. Cumulatively, around 39.1% had received at least *some* training. Of these, 31.7% received less than two hours, while 6% received up to four hours of training. Such training was planned in 4.8% of the facilities included in this survey.

### 3.5. Qualitative Findings

All free-text comments were subjected to inductive thematic statement analysis, identifying requirements at the micro, meso and macro level. These will be outlined in sequence below. At the micro-level, requirements related to *care recipients* were identified, while *organizational requirements* were identified at the meso level. At the macro level, requirements were related to *policy and legislation.* Finally, *technology requirements* will also be discussed, as prerequisites relating to the technology itself were identified across all three levels.

#### 3.5.1. Micro-Level: Care Recipient

Within the micro-level, three subthemes emerged; (i) user capabilities; (ii) user willingness; and (iii) family support. Comments related to *user capabilities* expressed the concern of not only the cognitive capabilities of the PWLD as users, but also their advanced age. In order to benefit from technology to promote social participation, respondents expressed that individuals need to have adequate experience and skills with technology. The inherent problem of older adults’ lower technology usage compared to the younger generations was expressed frequently. The respondents were generally pessimistic about elderly PLWD being able to utilize technology independently.

*“On average, they should be 30 years younger and open to digital technologies. They will be in 30 years.”*

(id_401)

Closely related to *user capabilities* is *user willingness,* as the respondents felt that beneficial outcomes through the usage of technology are also related to the willingness of the care recipient. Respondents emphasized openness and interest among care recipients to try novel technology. However, they concurrently expressed concern about many of their clients being quickly overwhelmed when operating technological devices.

*“They should have the ability to handle it. The currently cared-for seniors have not learned how to deal with today’s technologies, and most of them are not even willing to learn how to use them—they feel overwhelmed. It is still an absolute minority that uses digital technologies. Only the next generation of seniors will use digital technology because they are already using it today.”*

(id_1182)

In order to mitigate lacking capabilities or feelings of being overwhelmed, the presence of relatives that can support the care recipient is crucial from the respondents’ perspective. Family members are needed to actively participate with their relatives with healthcare needs in acquiring, getting familiar with, and using technology to help them stay connected. This implies that relatives also have the willingness and capabilities to support their loved ones in using such technology; two conditions requiring awareness of “what is out there” and potential benefits of use.

*“For people with dementia who live alone in their homes, I’m rather skeptical about such digital technologies because they have a view of the world that lies in the past. So, it would shake their worldview and also their self-image because it simply doesn’t fit into their world. Such technologies would only make sense if they were used together with caregivers.”*

(id_482)

#### 3.5.2. Meso-Level: Organizational Requirements

At the meso level, the organizational requirements could be divided into three parts: (i) technical support; (ii) training; and (iii) sufficient resources. When implementing novel technology, the need for someone that could be physically present to install and set up the system was emphasized. During the implementation phase, users of the technology, including care recipients, family members, and professional providers, need to receive appropriate instructions and support to avoid being overwhelmed and discouraged from engaging with the technology. However, *technical support* was expressed by respondents to be important not only during the implementation phase but also to be available continuously. Contact personnel exclusively handling technical issues was seen as a vital source of support that needs to be available to healthcare providers and care recipients and their family members.

*“Contact persons and people in charge who accompany the organization, administration and implementation, since nursing staff have too little time and knowledge of possible technologies and their application. For many clients, staff must be present during the entire period of use to provide support, which everyday life does not allow.”*

(id_337)

Goal-specific *training* and education were expressed as essential among many of the respondents. The need for training was voiced for service recipients, their family members, staff in healthcare organizations. This training needs to be tailored to the individuals’ needs to enable the independent use of technology and reduce the fear and anxiety that might arise when faced with new technology. This was especially important when training care recipients and their relatives. For healthcare providers, up-to-date education in technological solutions was described as an essential part of raising awareness of possible technological solutions and pedagogical training to support technology implementation.

*“Education (to take away the anxiety), instruction and accompaniment until the technology can either be safely operated by the user or an everyday helper who can provide support.”*

(id_1148)

The two statements above reflect the third requirement at the meso level, namely, *sufficient resources*. This requirement explicitly involved time and staff. As implementing new procedures and operating novel technology requires time, additional staffing was considered a key aspect. Whether this requirement was expressed as additional personnel for the familiarization process or trained staff specifically for operating the technology, the technology implementation requires more human resources. Furthermore, several collected comments expressed frustration over the inadequate time available to incorporate technology to effectively benefit providers and care recipients.

*“It would be great to have at least one additional job position in each nursing service/nursing home, financed through the nursing tariffs. This position should be specifically responsible for digital technologies and be able to train customers and employees. Overall, the introduction of the technologies would have to be supported more. We don’t get it done here because there is too little time left for it.”*

(id_195)

#### 3.5.3. Macro-Level: Policy and Legislation

At the macro level, the main requirements identified were related to (i) cost coverage; and (ii) network infrastructure. The issue of *cost coverage* was a recurring theme, where respondents urged for acquisition costs for hardware, software and internet connection to be covered. A consensus was shared among the survey participants that care recipients or service providers should not incur acquisition costs. Rather, technology promoting social health for people in need of care should be recognized as assistive technology and thereby be covered by the health insurer.

*“Secure communication channels, adequately fast and cheap internet, adequate equipment, possibly free Wi-Fi should be considered, additional rights to tablets for seniors with basic income support or welfare benefits.”*

(id_379)

A more permeating prerequisite that the respondents brought up was the issue of the *network infrastructure.* Many respondents expressed their frustration over the low internet broadband coverage, which hinders practical technology use, not only among care recipients but also among the healthcare providers. This issue was especially emphasized in rural communities, where some even struggled with proper cell phone reception.

*“Low-cost devices or cost support from health or long-term care insurers. Telecommunications providers must expand their offerings to explain and install this technology on-site. This responsibility must not be shifted to care employees.”*

(id_1246)

#### 3.5.4. Technology Requirements

The final requirement, reaching across all three levels, is found with the technology itself. These requirements were predominantly related to (i) availability; and (ii) user-friendliness. The requirement mentioned above at the macro-level, related to *network infrastructure,* will not be outlined again but serves to demonstrate the permeating requirements that technology has across all levels.

Within *availability*, respondents emphasized the need for available hardware, software and internet. In order to be able to connect with family, friends and healthcare providers, care recipients first need the required devices, with the appropriate software installed so that they are ready to use. Respondents emphasized that devices, such as touchscreen tablets, could improve the home environment and help stay in touch with healthcare providers. This necessitates proper internet connection opportunities within every household, a condition emphasized by respondents as frequently as required hardware. Many households in Germany, especially older adults, have analog telephone connections without internet, complicating the prospects of utilizing digital devices to connect beyond telephoning. As mentioned above, these connectivity issues concern private households and healthcare providers, especially in rural areas. Within this requirement, challenges with slow and unstable internet were often mentioned, severely hindering opportunities for care recipients and providers to develop towards a more technology-friendly healthcare provision.

Technology-friendly healthcare provision postulates *user*-friendly technology. Respondents urged for technology with simple usability and self-explanatory functions. For the technology to be appropriate for older adults, focused efforts need to be put into the design in terms of a large display and few buttons, possibly even offering a voice assistant function. This was especially emphasized among respondents caring for individuals with physical limitations. For individuals with cognitive impairments, conditions, such as limited application possibilities, were suggested to achieve simplicity of operation and favor visibility.

*“The technology must be available on site (Wi-Fi, laptop, camera, etc.), […], physical limitations must be taken into account (paralysis, etc.), the monitor must be large, and all buttons must be large and clearly arranged, possibly a voice assistant.”*

(id_1093)

## 4. Discussion

This paper aimed to describe the impact of the COVID-19 pandemic on PLWD in German nursing homes in terms of clinical outcomes, the availability of social activities, and the possibilities of using technology to enable social participation. Nearly 8200 German nursing homes were invited by email to provide information about the structural characteristics of their institution, the number of lab-confirmed COVID-19 cases in their nursing home, and the effect of the pandemic on clinical outcomes, such as BPSD and medication usage among their residents with dementia. Despite the high overall observed increase of BPSD, only 5.8% of the survey respondents saw an increase in the usage of pharmacological therapy for residents with dementia. The high occurrence of BPSD might be explained by the fact that nursing homes tend to care for residents with more severe stages of dementia [48,49,50], which research indicates is positively correlated with BPSD [44,45,51].

Another plausible explanation is the relatively high cancellation rate of social activities for care recipients (42.4%). Only 14.8% of the respondents reported procedures granting special access for visitors of residents with dementia in nursing homes. This is consistent with research showing a higher occurrence of BPSD in settings with low levels of social engagement and long durations of unoccupied time [8,23,30]. These restricted opportunities for social participation and increased social isolation might have contributed to the increased frequency of depression (38.9%), anxiety (38.6%) and appetite loss (24.1%).

A low proportion of survey respondents (6.5%) indicated that their facility had established procedures for using technology to promote social participation among PLWD. The majority of facilities did not have organized staff training to use technology to facilitate social participation among their residents. Six percent of all respondents had undergone half a day of training in using social technology, while barely one percent had undergone a whole day’s worth of training. Training of staff has been identified as a crucial prerequisite to the successful implementation of technology in long-term care facilities [52,53,54], which was echoed in the free-text comments made by the respondents.

Nevertheless, 72.8% had provided opportunities for residents with dementia to use digital communication technologies for social contact. In addition, a relatively high proportion had facilitated social participation for their residents with dementia using digital tools, such as music, videoconference tools and mobile applications. The long-term care setting might have enabled the high usage of technology for social purposes, as the nature of the care arrangements allows for consistent support and follow-up. The low number of respondents reporting increased use of pharmacological therapy might partially be attributed to the high uptake of technology-driven, non-pharmacological strategies to socially engage residents with dementia during COVID-19. However, due to the limitations of the data, this should only be considered as cautious speculation. Future research is warranted on the correlation between pharmacological therapies and psychosocial strategies for PWLD in nursing homes.

Some substantial barriers were identified in the qualitative component of this survey. The demands identified at the micro-, meso- and macro-level predominantly encompassed unmet prerequisites, posing considerable barriers. The requirements at the micro-level were directed at the care recipient in terms of *user capabilities*, *user willingness*, and *family support*. This finding is congruent with other studies looking into barriers and facilitators incorporating social technology in dementia caregiving [53,55,56]. The responses mostly expressed skepticism and had a pessimistic view on the likelihood of this generation’s older adults being able or willing to engage with technology to remain socially active when meeting in person is difficult. Without the availability of younger, cognitively healthy family members to support technology usage, respondents expressed that care recipients would too easily be overwhelmed or confused, thus impeding the potential benefits that such technology might provide. However, research indicates that the significantly lower adoption of new technology among older adults compared to younger generations [57] is not necessarily grounded in technology aversion [58], but rather the lack of sufficient support in doing so [55]. This truly highlights the importance of user-friendly technology adapted to individuals’ needs, one of the identified technology requirements.

Recommendations made by respondents also highlight the importance of sufficient resources at the meso-level. Not only in terms of time and staffing resources but also in resources to provide sufficient training and tech support. The outlined organizational requirements are essential to accommodate healthcare providers to mitigate some of the barriers outlined at the micro-level. Closely related to sufficient resources at the organizational level is *cost coverage*, identified as a part of the policy requirements at the macro-level. Sufficient resources require funding, implying the need for a digitalization strategy that recognizes the benefits of technology that promotes social health for care recipients. Similar shortcomings related to cost coverage of technological solutions have already been reported during the COVID-19 pandemic [59]. This also relates to the technology requirement of *availability*, especially in rural areas. The inadequate broadband infrastructure in Germany is a well-known and highly debated problem. Despite a high population density with more than 80 million citizens, Germany has been found to be way behind in broadband infrastructure [60]. Without the basic broadband coverage to reach homes and institutions, all other requirements are trivial. The basic condition that needs to be met to allow users to stay connected requires the German *network infrastructure* to be expanded, with *fiber* optic cables, on a massive scale.

### Limitations

The results reported above are based on an opportunity sample of 417 nursing homes from all federal states, representing about 3.7% of Germany’s approximately 11,300 full inpatient nursing homes [61]. Although a comparison of the sample with national averages concerning structural characteristics indicates no structural bias, self-selection and a corresponding bias cannot be ruled out. One major limitation in this study is that information is mainly collected from facility managers and directors of nursing of German nursing homes and their reported observations of their residents with dementia. Without patient-specific characteristics, such as gender, age, cognitive abilities and comorbidities, statistical analyses of correlations between residents’ clinical outcomes and other variables cannot be conducted. As this study is of an explorative, descriptive nature, further research, including data on patient characteristics and nursing outcomes, is needed. Furthermore, the survey did not contain question items about the preferences of *residents* in using any of the digital devices in social activities, something which might limit a deeper and nuanced understanding of the contextual factors influencing the opportunities to use technology for social purposes. Despite evidence to suggest that technology can foster social health in PLWD by facilitating social participation and alleviating isolation [62,63,64], more research is warranted to assess the usability and acceptance of technology among nursing home residents with dementia. Another limitation to consider is that the aspects concerning the social health of PLWD specifically (and the role of technology in doing so) stem from survey items that were only included in the follow-up study of the impact of COVID-19 on German nursing facilities. This resulted in fewer responses involving these aspects, making the description less nuanced. The retrospective nature of the questions included in this survey may have led to recollection bias among the respondents regarding the observed increase of BPSD and pharmacological treatment. Results should therefore be interpreted with caution. Finally, the results presented here are time-sensitive; thus, shifts may have already occurred in the period since the survey.

## 5. Conclusions

During and beyond the COVID-19 pandemic, non-pharmacological treatments, such as technology-driven solutions to promote social health among nursing home residents with or without dementia, should be an integrated part of caregiving procedures. The high increase in depression and anxiety among PLWD observed during the pandemic might be an indication of unmet psychosocial needs caused by the high frequency of discontinued social activities for residents with dementia. Participation in meaningful activities is an integral part of social health, meaning that the abrupt suspension of social activities for persons in need of care, with or without dementia, can significantly impact clinical outcomes for this population. Considering what we know of the adverse consequences of social isolation, keeping non-pharmacological options available, such as psychosocial interventions, is imminent in disruptive events such as a viral outbreak. As found in our study, a staff shortage of only five percent (and higher) was significantly correlated with social activities for residents with dementia being canceled, revealing how easily neglectable social health interventions/strategies for nursing home residents are. Although more than 70% of the respondents reported having provided opportunities for residents with dementia to use technology for social purposes, the low frequency of established procedures seems to indicate the implementation of ad hoc solutions to safeguard the social health of residents with dementia. These findings imply that technology should be incorporated as standard offers to maintain social participation among persons in need of care with or without dementia. This does not imply that technology should be implemented only due to the lack of such, but in a person-centered manner, guided by residents’ preferences. This, in turn, demands established guidelines and ongoing training of staff. However, developing and implementing technology to promote social participation faces substantial barriers as long as social health is not recognized on equal terms as the physical and mental health domains. Acknowledging social health as a priority before we can implement technological solutions to promote this health domain requires spearheaded efforts at the societal-, organizational- and individual levels.

## Figures and Tables

**Table 1:** Summary of findings according to facility type.

Variables (N ^a^)	N ^b^ (SD)	% ^c^
Sector (N = 401)		
Public	37	9.2
Private	149	37.2
Non-Profit	215	53.6
Special dementia care contract (N = 407)	70	17.2
Average no. of healthcare staff per facility (SD) (N = 366)	48.3 (26.5)	-
Average client capacity per facility (SD) (N = 404)	86.3 (41.2)	-
Confirmed COVID-19 cases among residents ($\overset{-}{x}$ ^d^) (N = 402)	212 (21)	52.7
Confirmed COVID-19 cases among staff ($\overset{-}{x}$) (N = 402)	281 (11)	69.9
Average no. of deaths with COVID-19 among residents (N = 139)	7 (6.61)	-
Social activities canceled (N = 366)	155	42.4
Special access to visit residents with dementia (N = 284)	42	14.8
Established procedures to use technology with residents with dementia (N = 369)	24	6.5
Opportunities to use digital communication technology for social contact (N = 349)	254	72.8
Social Tech training for staff (N = 353)		
None	179	50.7
Less than 2 h	112	31.7.
Up to 4 h	21	6.0
Up to 8 h	4	1.1
Over days	1	0.3
Training is planned	17	4.8
Observed increase of pharmacological therapy (N = 344)	20	5.8
Observed increase of BPSD ^e^ (N = 373)		
Aggression	63	16.9
Anxiety	144	38.6
Apathy	60	16.1
Appetite loss	90	24.1
Depression	145	38.9
Hallucinations	5	1.3
Paranoia	2	0.5
Psychosis	17	4.6
Sleeplessness	39	10.5
Wandering	63	16.9
Other	36	9.7

^a^ N = valid responses per question item. ^b^ Numbers do not add up to total number of survey participants due to missing values excluded for each question item. ^c^ Percentage reported according to relative frequencies. ^d^ Average across facilities with confirmed cases of COVID-19. ^e^ BPSD = Behavioral and Psychological Symptoms in Dementia.

**Table 2:** Association between structural characteristics and social activities for PLWD being canceled.

			Social Activities Canceled		Total	Χ^2^	*p*
			Yes	No			
type of provider	public	N	15	17	32	3.5929	0.464
		%	46.9	53.1	100		
	private	N	47	81	128		
		%	36.7	63.3	100		
	non-profit	N	87	100	187		
		%	46.5	53.5	100		
	total	N	149	198	347		
		%	42.9	57.1	100		
special dementia care contract	yes	N	26	37	63	1.3689	0.504
		%	41.3	58.7	100		
	no	N	129	168	297		
		%	43.4	56.6	100		
	total	N	155	205	360		
		%	43.1	56.9	100		
cases among residents	yes	N	93	98	191	7.693	0.021 *
		%	48.7	51.3	100		
	no	N	53	102	155		
		%	34.2	65.8	100		
	total	N	146	200	346		
		%	42.2	57.8	100		
cases among staff	yes	N	119	133	252	9.9753	0.007 **
		%	47.2	52.8	100		
	no	N	27	67	94		
		%	28.7	71.3	100		
	total	N	146	200	346		
		%	42.2	57.8	100		
>5% staff shortage	yes	N	101	96	197	13.0971	0.001 **
		%	51.3	48.7	100		
	no	N	53	107	160		
		%	33.1	66.9	100		
	total	N	154	203	357		
		%	43.1	56.9	100		

* Significant at 5 % level. ** Significant at 1 % level.

## Data Availability

The data presented in this study are not publicly available due to privacy regulations. A translated excerpt of the survey containing the question items subjected to analysis for the purpose of this study is available as Supplementary Materials.

## Supplementary Materials

The following supporting information can be downloaded at: https://www.mdpi.com/article/10.3390/ijerph19041956/s1, S1: Questionnaire Excerpt.
