# Supplementary material for: Social Health among German Nursing Home Residents with Dementia during the COVID-19 Pandemic, and the Role of Technology to Promote Social Participation
Source: Int J Environ Res Public Health. 2022 Feb 10;19(4):1956. doi: 10.3390/ijerph19041956 (PMC8872488; doi:10.3390/ijerph19041956)
Supplement: Supplementary file 1 [file ijerph-19-01956-s001.zip › ijerph-1508445-supplementary.pdf]

**1 Introductory text facilities**

*[…] We refer to the persons cared for in or by the facility as persons in need of care/clients. We refer to nursing services, nursing homes and day care as facilities.*

**2 Details of the facility**

In what kind of facility are you working?
*Please select one. If you work for more than one facility, please choose the type of facility for which you currently evaluate the greatest need for support during the pandemic.*

- o In outpatient care/ in a nursing service
- o In inpatient long-term care/ in a nursing home
- o In semi-inpatient care/ in short-term care/ in a day clinic
- o In another type of facility

In which federal state is your facility located?

- o Baden-Wuerttemberg
- o Bavaria
- o Berlin
- o Brandenburg
- o Bremen
- o Hamburg
- o Hessen
- o Mecklenburg-Western Pomerania
- o Lower Saxony
- o North Rhine-Westphalia
- o Rhineland-Palatinate
- o Saarland
- o Saxony
- o Saxony-Anhalt
- o Schleswig-Holstein
- o Thuringia

What is the sponsorship of your facility?

- o public facility
- o non-profit facility
- o private provider

**2.1 Function of participant**

What is your function in the facility?
*Multiple selection possible.*

- ☐ Facility management
- ☐ Directors of nursing (Nursing service manager/ responsible professional staff)
- ☐ Quality Manager

**2.2 Standard page**

Does your facility have a focus on nursing care of people with dementia agreed in the care contract?

- o yes

- ○ no, another focus agreed in the health care contract
- ○ no, no focus agreed in the health care contract
- ○ not reported

**3 Entry block 2**

*Now we ask you to provide information on the occurrence of the SARS-CoV2 virus in your facility and your experience with illnesses or suspected cases among the persons in need of care/clients and employees.*

**4 COVID in facility**

Are there or were there confirmed COVID-19 cases among the care recipients you care for?

- ○ no
- ○ yes

if yes, how many to the present date? Please enter the number.

[ ]

How many of the persons in need of care with COVID-19 disease have died to date?

[ ]

How many of the persons in need of care with COVID-19 disease have died since October 2020?

[ ]

**5 COVID Personnel**

Are there or were there confirmed COVID-19 cases among the employees?

- ○ no
- ○ yes

if yes, how many to the present date? Please enter the number.

[ ]

How high was the staff shortage in your facility due to the COVID 19 pandemic (including possible quarantine measures) in December 2020?

- ○ no shortage
- ○ < 5%
- ○ 5 - <10%
- ○ 10 - <20%
- ○ 20 - <30%
- ○ 30- <40%
- ○ 40- <50%
- ○ 50% and more
- ○ not reported

**6 Participation in social activities for people living with dementia**

At the current time, are there any special arrangements in terms of access restrictions to visit…
*Multiple selection possible.*

- ☐ People with dementia

☐ Terminally ill people
☐ People with psychiatric diseases
☐ People after acute events, e.g. accidents, illnesses
☐ Other people in need of care, namely:

In the last six months: Have offers of social activities for residents with dementia been cancelled/ discontinued?

- no
- yes
- non-applicable
- not reported

**7 Clinical conditions in people living with dementia**

In the last six months: Have you noticed an increase in neuropsychiatric symptoms among care recipients with dementia as a result of the pandemic?
*Multiple selection possible.*

☐ Agitation/aggression
☐ Anxiety
☐ Apathy
☐ Depression
☐ Increased motor activity (walking tendencies, wandering)
☐ Hallucination
☐ Loss of appetite
☐ Paranoia
☐ Psychotic episodes
☐ Insomnia
☐ Other symptoms, namely:

In the past six months: Have you noticed an increase in pharmacological therapies for people living with dementia as a result of the pandemic, such as an increase in prescribed antipsychotics, sedatives, or antihypnotics?

- no
- yes
- non-applicable
- not reported

**8 Use of technology for social purposes with people living with dementia**

During the pandemic, did you create additional opportunities for care recipients with dementia to use digital communication technologies to socialize with friends, family, or others?

- yes, the following:
- no, but we are planning to
- no
- not reported

Are there concepts in the facility that support the use of digital technologies for the social participation and activation of people in need of care?
*By a concept, we mean recommendations for action, guidelines, or agreements that describe the procedure, frequency, duration and evaluation of the use of a digital technology to support social participation and activation.*

- There is a general concept on the use of technology
- There are one or more target group-specific concepts for the use of technology
- There is a specific concept for the use of technology together with people with dementia
- The development of a concept for the use of technology is planned
- There is no concept for the use of technology
- non-applicable
- not reported

Was there training for nursing staff during the pandemic to accompany the use of digital technologies to promote social participation and activation?

- yes, duration under 2 hours
- yes, duration between 2 and 4 hours
- yes, duration between 4 and 8 hours
- yes, several days duration
- no, but is planned
- no
- non-applicable
- not reported

Does the facility use digital technologies to engage and activate people with dementia in social activities?
*This refers to digital care and activity services, digital services for exchanging and communicating with other people, or digital services for training physical or cognitive skills.*
*Multiple selection possible.*

Videoconference tools (Skype, Facetime etc.)

- yes, used prior to the pandemic as well
- yes, introduced after the start of the pandemic
- no, but is planned
- no

Digital music therapy

- yes, used prior to the pandemic as well
- yes, introduced after the start of the pandemic
- no, but is planned
- no

Mobile applications (apps) e.g. cognitive activation

- yes, used prior to the pandemic as well
- yes, introduced after the start of the pandemic
- no, but is planned
- no

Social robots

- ○ yes, used prior to the pandemic as well
- ○ yes, introduced after the start of the pandemic
- ○ no, but is planned
- ○ no

Video games (e.g., video consoles)

- ○ yes, used prior to the pandemic as well
- ○ yes, introduced after the start of the pandemic
- ○ no, but is planned
- ○ no

Virtual Reality (VR-headsets)

- ○ yes, used prior to the pandemic as well
- ○ yes, introduced after the start of the pandemic
- ○ no, but is planned
- ○ no

Others, namely:

- ○ yes, used prior to the pandemic as well
- ○ yes, introduced after the start of the pandemic
- ○ no, but is planned
- ○ no

What conditions are necessary for people in need of care to be able to use digital technologies for social participation, activation and communication?
*Please describe.*
